# Biofortification of Broccoli Microgreens (*Brassica oleracea* var. *italica*) with Glucosinolates, Zinc, and Iron through the Combined Application of Bio- and Nanofertilizers

**DOI:** 10.3390/foods12203826

**Published:** 2023-10-19

**Authors:** Carlos Esteban Guardiola-Márquez, C. Valentina García-Sánchez, Óscar Armando Sánchez-Arellano, Erika Melissa Bojorquez-Rodríguez, Daniel A. Jacobo-Velázquez

**Affiliations:** 1 Tecnologico de Monterrey, Escuela de Ingenieria y Ciencias, Ave. General Ramon Corona 2514, Zapopan 45138, Jalisco, Mexico; a01560073@tec.mx (C.E.G.-M.);; 2 Tecnologico de Monterrey, Institute for Obesity Research, Ave. General Ramon Corona 2514, Zapopan 45201, Jalisco, Mexico

**Keywords:** beneficial microorganisms, nanoparticles, micronutrient deficiency, mineral fertilization, plant nutritional quality, plant growth-promoting microorganisms, plant nutrition

## Abstract

There is a severe need to develop a sustainable, affordable, and nutritious food supply system. Broccoli microgreens have attracted attention due to their rich nutritional content and abundant bioactive compounds, constituting an important opportunity to feed the ever-increasing population and fight global health problems. This study aimed to measure the impact of the combined application of biofertilizers and zinc and iron nanofertilizers on plant growth and the biofortification of glucosinolates (GLSs) and micronutrients in broccoli microgreens. Biofertilizers were based on plant growth-promoting (PGP) bacterial consortia previously isolated and characterized for multiple PGP traits. Nanofertilizers consisted of ZnO (77 nm) and γ-Fe_2_O_3_ (68 nm) nanoparticles synthesized with the coprecipitation method and functionalized with a *Pseudomonas* species preparation. Treatments were evaluated under seedbed conditions. Plant growth parameters of plant height (37.0–59.8%), leaf diameter (57.6–81.1%) and fresh weight (112.1–178.0%), as well as zinc (122.19–363.41%) and iron contents (55.19–161.57%), were mainly increased by nanoparticles subjected to the functionalization process with *Pseudomonas* species and uncapped NPs applied together with the biofertilizer treatment. Regarding GLSs, eight compounds were detected as being most positively influenced by these treatments. This work demonstrated the synergistic interactions of applying ZnO and γ-Fe_2_O_3_ nanofertilizers combined with biofertilizers to enhance plant growth and biofortify micronutrients and glucosinolates in broccoli microgreens.

## 1. Introduction

Currently, regulations and actions regarding food security are mainly aimed at ensuring food quantity and daily calorie intake rather than improving food quality [1]. Modern agricultural practices such as overfertilization, excessive land use, crop intensification, the use of high-yielding varieties, and poor micronutrient fertilization are progressively producing nutrient-deficient foods and contributing significantly to alarming problems through public health, soil degradation, and environmental damage of agroecosystems [1,2,3,4,5]. Modern agriculture is increasingly linked with micronutrient deficiencies in the population, including iron and zinc, which correspond to the most widespread micronutrient deficiencies worldwide [1,6]. In addition, increased consumption of low-nutrient, cheap, energy-dense, and processed foods have contributed significantly to the current alarming rates of malnutrition and other chronic diseases [7,8].

There is a serious need to develop more sustainable, affordable, accessible, and nutritious food products [9]. In this sense, the use of microgreens has gained great interest and popularity in recent years [10]. Microgreens are seedlings of edible plants that are harvested after cotyledonary leaves are developed, approximately 7–14 days after germination [9,11,12]. They are versatile crops that can be cultivated easily, quickly, and cost-effectively and that are eco-friendly due to the simple requirements for supplies and equipment, making them an important opportunity for dietary supplementation [9,10]. Microscale vegetables are reported to contain lower concentrations of antinutrients and higher amounts of nutrients and bioactive compounds such as amino acids, simple sugars, minerals (e.g., zinc and copper), carotenoids, phenolic compounds, and vitamin C, compared with their mature forms [7,9,12]. These compounds come from the secondary metabolism and the enzymatic breakdown of macromolecules [10].

Plant species from the *Brassicaceae* family are among the most used for sprouting purposes and, thus, are among the most reported in the scientific literature [10,11]. Particularly, broccoli (*Brassica oleracea* var. *italica*) microgreens have attracted attention due to their rich nutritional content and abundant bioactive compounds, especially glucosinolates (GLSs) [11,12,13,14,15]. Bhandari et al. [16] reported that broccoli contains the highest total glucosinolates in sprouts (162.19 µmol·g^−1^) and seeds (110.76 µmol·g^−1^) compared with other *Brassica* species, including cabbage, radish, cauliflower, pakchoi, baemuchae, leaf mustard, and kale. Studies have shown that consuming broccoli microgreens, highly concentrated in GLSs, can reduce the incidence of colon, colorectal, bladder, and lung cancers; significantly relieve type 2 diabetes symptoms (such as insulin resistance and oxidative stress); protect against *Helicobacter pylori* infections, brain injuries, and light-induced damage of the retina; and exert anti-obesogenic effects [10,11,17,18,19]. Much of the bioactivity and health benefits of broccoli come from the products of the hydrolysis of glucosinolates [12]. Glucoraphanin (GRA) is considered one of the most relevant GSLs due to its anticancer properties. Researchers are interested in improving the GRA content in *Brassica* species due to its health-promoting potential [12,20,21,22]. Phenylethyl isothiocyanate hydrolyzed from gluconasturtiin (GNS) have exhibited antimicrobial and anti-cancer properties against colon and prostate cancer [20]. Recent research has also shown relevant in vivo and in vitro assays regarding GLSs’ anti-obesogenic potential, they participate in the reduction of circulating lipopolysaccharides (LPS), weight gain, obesity-induced inflammation, and lipid accumulation; modulation of hypercholesterolemia and gut microbiota; and enhancing insulin sensitivity and white fat browning [9,11,12,17]. In contrast, progoitrin (PRO) is considered not only harmful to human health, but is also responsible for the bitter flavor of *Brassica* vegetables [12].

Interestingly, stressful conditions during germination can stimulate secondary metabolism and increase the content of relevant phytochemicals such as GLSs of microgreens [10]. Also, nutrient fortification can significantly influence the metabolic activities of microgreens and enhance their growth and nutritional quality [9]. This process of improving the nutritional quality of food crops during their cultivation is called biofortification [1,8,23,24], which is an important strategy to enrich macro and micronutrients, as well as bioactive compounds in the edible parts of plants [1,7,24,25].

Bio-fortification through bio and nanofertilization are promising strategies due to their sustainability, efficiency, cost-effectiveness, and low environmental impact. In addition, these fertilization methods enhance the efficiency of agricultural inputs, contributing to generating sustainable agroecosystems [24,26]. Biofertilizers are formulations of plant growth-promoting microorganisms (PGPM) that establish symbiotic relationships with plants and can promote their growth and enrich their nutritional value by stimulating plant metabolism and enhancing nutrient uptake through multiple mechanisms including the mobilization of macro and micronutrients (mainly by P, K, and Zn solubilization; nitrogen fixation; and siderophores production), secretion of plant growth regulators (including phytohormones and organic acids), abiotic stress resistance, and plant protection against phytopathogens [2,24,25,27,28]. Nanofertilizers consist of nanoscale nutrients (1–100 nm) that present unique physicochemical properties, including a high surface area to volume ratio and increased reactivity and functionalization properties, compared with their bulk macrostructure counterparts (commonly used as conventional mineral fertilizers). The nanoscale improves plant nutrient absorption, transport, and utilization, facilitating their penetration through biological barriers, diffusion to the vascular system, and assimilation in the different plant organs [28,29,30,31]. Nanofertilizers have been reported to be between 20 to 30% more efficient than conventional mineral fertilizers [28,32,33,34]. This work involves zinc and iron nanofertilization as these micronutrients are highly deficient in the global population; around 20–25% and 17.3% of the population are Fe and Zn deficient, respectively. Fe and Zn are required for several critical functions, including protein synthesis, enzymatic function, DNA replication, cognition, immune response, reactive oxygen species detoxification and antioxidant activity, and the alleviation of chronic diseases [6,25]. Both strategies of bio and nanofertilization have been successfully applied in microgreens to improve the nutritional and phytochemical content [7,13,35,36].

Microgreens have been extensively investigated as new functional foods or nutraceuticals that are beneficial to human health [10], which make them an excellent target for biofortification assays [9]. Therefore, this study aimed to measure the impact of the combined application of biofertilizers of PGPR and zinc and iron nanofertilizers on plant growth and biofortification with glucosinolates and micronutrients in broccoli microgreens grown under seedbed conditions.

## 2. Materials and Methods

### 2.1. Chemicals and Plant Materials

Iron (II) sulfate heptahydrate (FeSO_4_·7H_2_O) and ferric chloride (FeCl_3_·6H_2_O) were obtained from J.T. Baker Chemical Co. (Phillipsburg, NJ, USA). Zinc acetate dihydrate (ZnC_4_H_6_O_4_·2H_2_O), sodium acetate (CH3COONa), sinigrin hydrate, sulfatase from *Helix pomatia*, diethylaminoethyl (DEAE)-sephadex A-25, acetonitrile (HPLC grade), methanol (HPLC grade), and ethanol (HPLC grade) were purchased from Sigma-Aldrich (Saint Louis, MO, USA). Desulfoglucoraphanin was obtained from Santa Cruz Biotechnology (Dallas, TX, USA). Deionized water (18.2 MΩ·cm resistance) used in the protocols was acquired from a Milli-Q Element water purification system (Millipore, Bedford, MA, USA). Commercial broccoli (*Brassica oleracea* L. var. *italica*) seeds Vita^®^ were obtained from “Rancho Los Molinos” company (Tepoztlan, Mexico).

### 2.2. Formulation of Biofertilizers Based on Native Plant Growth-Promoting Microorganisms

**Figure 1:**
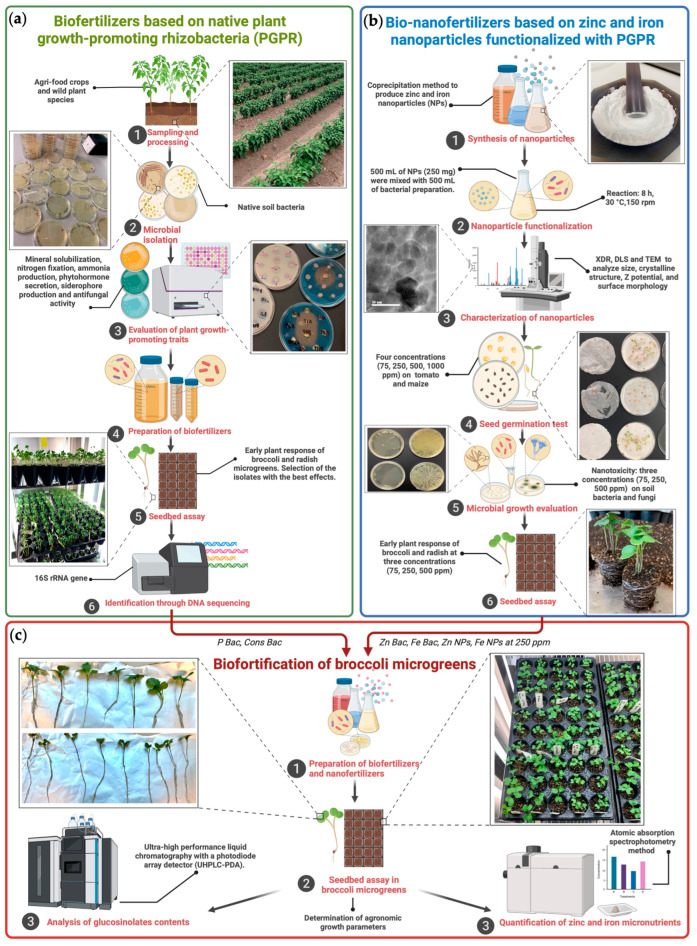
Complete experimental strategy considering the formulation of biofertilizers based on plant growth-promoting rhizobacteria (**a**) as reported in Guardiola-Márquez et al. [27]; the production and evaluation of zinc and iron bio-nanofertilizers (**b**) described by Guardiola-Márquez et al. [39]; and the biofortification of broccoli microgreens with glucosinolates, zinc, and iron using the bio- and nanoformulations with the best performance in previous works (**c**). Zn Bac, bacteria functionalized ZnO NPs. Fe Bac, bacteria capped γ-Fe_2_O_3_-NPs. Cons Bac, bacterial consortia used as a capping agent. Zn NPs, uncapped ZnO NPs. Fe NPs, uncapped γ-Fe_2_O_3_-NPs. P Bac, phosphate-solubilizing bacterial biofertilizer. Figure created with BioRender.com.

Biofertilizers were based on plant growth-promoting bacteria that were previously isolated and characterized as described in Guardiola-Márquez et al. [27]. Briefly, native bacterial strains were isolated from agri-food crops and wild plant species in northern Mexico and then characterized for several plant growth-promoting (PGP) traits (including potassium, phosphate, and zinc solubilization; nitrogen fixation; ammonia production; indole-3-acetic acid (IAA) secretion; siderophore production; and antifungal activity against phytopathogenic *Fusarium oxysporum)* and finally evaluated on radish and broccoli microgreens to select potential biofertilization agents. In this earlier work, bacteria were grouped depending on their relevance for each PGP attribute. The consortia named “P bac” was used in the present study as it was one of the treatments with the best performance to promote seedling growth and was formulated with bacterial isolates showing multiple PGP traits at high levels, mainly mineral solubilization. Details regarding PGP traits’ levels of each bacterial isolate and results on the early plant response evaluation are also shown in Guardiola-Márquez et al. [27] (Figure 1a).

The “P bac” consortia consisted of *Serratia liquefaciens* strain A302, *Pseudomonas extremorientalis* strain A306A1, *Pseudescherichia vulneris* strain A334, and *Serratia liquefaciens* strain B14FEB, which were placed individually in 40 mL of sterile trypticase soy broth (TSB) and incubated on a rotary shaker at 180 rpm and 30 °C for 48 h. Bacterial cell density was monitored spectroscopically with a microplate reader VARIOSKAN LUX (ThermoFisher Scientific, Waltham, MA, USA) until obtaining optical density values at 600 nm (OD_600_) between 0.6 and 0.8, which corresponded to total plate counts of 1.2 × 10^8^–1.6 × 10^8^ CFU mL^−1^ [37,38]. Bacterial growth was also estimated by plate count in trypticase soy agar (TSA) plates [27].

### 2.3. Preparation of Zinc and Iron Nanofertilizer

The synthesis and characterization process of the zinc and iron nanoparticles used in this study were previously reported in Guardiola-Márquez et al. [39]. A co-precipitation method was used to synthesize hexagonal wurtzite ZnO nanoparticles (ZnO NPs; 76.84 ± 10.3 nm) from 0.5 M zinc acetate dihydrate (ZnC_4_H_6_O_4_·2H_2_O) solution as the starting material, and face-centered cubic maghemite (γ-Fe_2_O_3_-NPs; 67.7 ± 8.9 nm) using 0.3 M iron (II) sulfate heptahydrate (FeSO_4_·7H_2_O) and 0.6 M ferric chloride (FeCl_3_·6H_2_O) as the precursor solution for iron nanoparticles. Nanoparticles were subjected to a functionalization process to incorporate surface capping; for this purpose, a bacterial consortium of native *Pseudomonas* species was used. Bacterial species corresponded to four *Pseudomonas* species (*Pseudomonas allii* strain B5KEA, *Pseudomonas marginalis* strain B9M, *Pseudomonas protegens* strain A276, and *Pseudomonas sesami* strain A137A1) that were earlier isolated, characterized for several PGP traits and identified through DNA sequencing [27]. Further details of the NP functionalization and characterization process are presented in Guardiola-Márquez et al. [39]. Bacteria capped ZnO and γ-Fe_2_O_3_ NPs at 250 ppm were used in the present work as they presented the bio-nanofertilizing potential in the previous in vivo assays in broccoli and radish (Figure 1b) [39].

### 2.4. Evaluation of Bio- and Nanoformulations in Broccoli Microgreens

A seedbed experiment was performed for 12 days to analyze the biofortification potential of the bio- and nanoformulations (Figure 1c); a completely randomized design was used. Twelve treatments were tested: (1) Bacteria functionalized ZnO NPs at 250 ppm (Zn Bac). (2) Bacteria capped γ-Fe_2_O_3_ NPs at 250 ppm (Fe Bac). (3) Bacterial consortia used as NP capping agents (Cons Bac). (4) Uncapped ZnO NPs at 250 ppm (Zn NPs). (5) Uncapped γ-Fe_2_O_3_ NPs at 250 ppm (Fe NPs). (6) Mineral ZnO NP precursor (Zn Prec). (7) Mineral γ-Fe_2_O_3_ NP precursor (Fe Prec). (8) P-solubilizing bacterial biofertilizer (P Bac). (9) Application of treatments 4 and 8. (10) Application of treatments 5 and 8. (11) Application of treatments 6 and 8. (12) Application of treatments 7 and 8. Negative control was untreated plants irrigated with water (− control). Three cavities (replicates) were evaluated per treatment. The assay was performed in plastic seedbeds of 72 cavities (5 × 5 × 6 cm), filled to ¾ of their capacity with sterile black soil mixed with vermiculite (3:1 *v*/*v*) [27,39].

Commercial broccoli (*Brassica oleracea* L. var. *italica*) seeds were surface sterilized by soaking in 70% ethanol for 30 s, followed by 5% sodium hypochlorite solution for 5 min, and five washes with sterile water [40]. The seeds were soaked in sterile distilled water with aeration overnight at room temperature to induce germination. Five seeds were sown per cavity at 0.5–1 cm depth and thinned to three plants per pot after germination [37]. Nine plants were tested per treatment. The seedbeds were watered daily with tap water. Plants were grown in a growth chamber at 25 °C and 70% relative humidity under a 16 h:8 h light/dark cycle [27,39].

Bio- and nanofertilization treatments were applied three times during the experiment at days 1, 6, and 9, applying 2 mL of the formulations to the corresponding cavities of each treatment. Experimental plants were harvested after 12 days and measured for agronomic parameters; microgreens were then immediately frozen with liquid nitrogen and stored at −80 °C (Revco Ultima PLUS ULT1386, Thermo Scientific Inc., Waltham, MA, USA) [27,39]. Frozen samples were freeze-dried at −83 °C and 0.035 mbar for 72 h (Labconco, Kansas City, MO, USA), lyophilized seedlings were ground to a fine powder and stored at −80 °C for further analysis [41,42].

#### 2.4.1. Determination of Plant Growth Parameters

Agronomic growth parameters of plant height (cm), leaf diameter (cm), root length (cm), and shoot fresh weight were measured. Plant height was determined from the base to the tip of the plants [27,39,43].

#### 2.4.2. Analysis of Glucosinolates Contents

The extraction and desulfation of GSLs from broccoli microgreens were performed as previously described [22]. To extract glucosinolates, 0.2 g of lyophilized broccoli microgreens powder was added with 10 mL of a pre-heated (10 min at 70 °C in a water bath (VWR, Radnor, PA, USA) ethanol/water (50:50, *v*/*v*) solution, followed by the addition of 50 µL of a 3 mM solution of sinigrin as the internal standard (I.S). Suspensions were incubated for 1 h at 250 rpm and 40 °C in a shaking incubator. The extracts were removed from the incubator, left to cool at room temperature, and then centrifuged (SL16R Thermo Scientific, Bremen, Germany) at 18,000× *g* for 10 min and at 4 °C. The supernatant was recovered and stored at −80 °C for further glucosinolates analysis.

GSLs were desulphated and purified using disposable polypropylene columns (Thermo Fisher Scientific, Waltham, MA, USA). The columns were prepared by adding 0.5 mL of water, followed by 0.5 mL of previously prepared resin Sephadex A-25 and an additional 0.5 mL of HPLC water. Then, 3 mL of broccoli extract supernatant was added to the prepared column and allowed to elute slowly. After removing the excess supernatant, the columns were washed with 2 × 0.5 mL of HPLC water followed by 2 × 0.5 mL of 0.02 M sodium acetate. Then, 75 μL of purified sulfatase previously prepared was added to each sample and left at room temperature overnight (12 h). Desulfoglucosinolates were eluted in vials with a total of 1.25 mL of water (2 × 0.5 mL + 0.25 mL) [44,45].

Glucosinolates were assayed using ultra-high performance liquid chromatography with a photodiode array detector (UHPLC-PDA). Individual GSLs were prepared using a standard curve of desulfoglucoraphanin ranging from 0 to 1250 ppm. The concentrations of total and individual GSL were expressed as mg of desulfoglucoraphanin equivalents per g of broccoli microgreens dry weight (DW), while individual GSL were identified based on retention time compared with authentic standards and related published data [45,46,47]. Chromatographic separations were performed on a UHPLC-PDA Acquity Arc system (Waters, Milford, MA, USA). Desulfoglucosinolates were separated on a Waters Cortecs reverse phase C18 (4.6 × 50 mm, 2.7 μm pore size) column using water (phase A) and acetonitrile (phase B) as mobile phases with a flow rate of 1.5 mL/min and a sequential gradient of 0/100, 28/80, and 35/100 (min/% phase A). The injection volume was 20 μL and the compounds were detected at 227 nm [22,45].

#### 2.4.3. Quantification of Zinc and Iron Micronutrients

The lyophilized microgreen powder was analyzed for the content of zinc and iron using the atomic absorption spectrophotometry method [14,48]. Quantification was performed with a PinAAcle 900F Atomic absorption (AA) spectrometer (Perkin Elmer, Waltham, MA, USA). The results were expressed as mg/100 g of dried plant material.

#### 2.4.4. Statistical Analysis

Statistical analyses of the experimental data were performed using one-way analysis of variance ANOVA and Tukey Test (*p* < 0.05) to compare mean values. Three replicates were considered, and the data represent the mean values ± standard deviation (SD). Jmp software version 17.0 (SAS Institute Inc., Cary, NC, USA) was employed for statistical analysis [22,27,39].

## 3. Results

### 3.1. Effects of Bio- and Nanoformulations on the Growth of Broccoli Microgreens

Twelve treatments, including biofertilizers and nanofertilizers, were tested in a 12-day seedbed assay in broccoli (*Brassica oleracea* L. var. *italica*) microgreens. To identify their impact on the plant yield, the agronomic growth parameters of plant height, leaf diameter, root length, and shoot fresh weight were measured.

**Figure 2:**
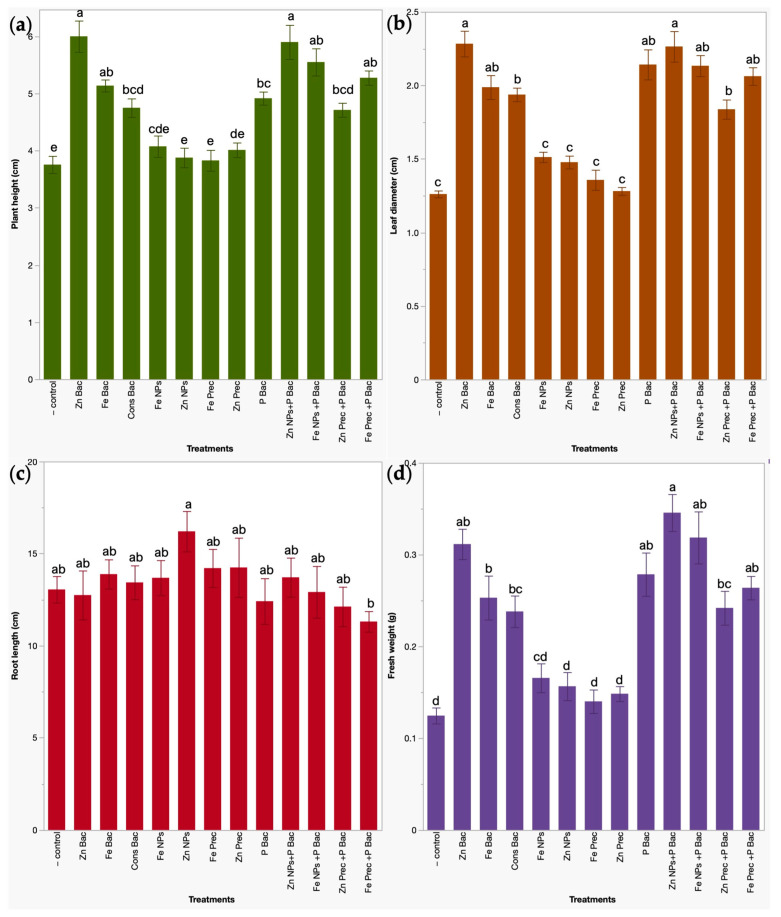
Evaluation of bio- and nanoformulations on agronomic parameters in broccoli microgreens. (**a**) Plant height (cm). (**b**) Leaf diameter (cm). (**c**) Root length (cm). (**d**) Fresh weight (g). Results correspond to mean ± standard deviation; letters indicate significant differences between treatments (*p* < 0.05). − control, water irrigated plants. Zn Bac, bacteria functionalized ZnO NPs. Fe Bac, bacteria capped γ-Fe_2_O_3_ NPs. Cons Bac, bacterial consortia used as the capping agent. Zn NPs, uncapped ZnO NPs. Fe NPs, uncapped γ-Fe_2_O_3_ NPs. Zn Prec, mineral ZnO NP precursor. Fe Prec, mineral γ-Fe_2_O_3_ NP precursor. P Bac, phosphate-solubilizing bacterial biofertilizer. Zn NPs + P Bac, uncapped ZnO NPs applied with the biofertilizer. Fe NPs + P Bac; uncapped γ-Fe_2_O_3_ NPs applied in conjunction with biofertilizer. Zn Prec + P Bac, mineral ZnO NP precursor applied with biofertilizers. Fe Prec + P Bac, mineral γ-Fe_2_O_3_ NP precursor applied in combination with biofertilizer.

Regarding plant height, the most relevant treatments were bacteria functionalized ZnO NPs (Zn Bac), uncapped ZnO NPs applied in conjunction with the biofertilizer treatment (Zn NPs + P Bac), uncapped γ-Fe_2_O_3_ NPs applied with biofertilizer (Fe NPs + P Bac), mineral γ-Fe_2_O_3_ NP precursor applied in combination with biofertilizer (Fe Prec + P Bac), and bacteria capped γ-Fe_2_O_3_ NPs (Fe Bac). Treatments containing zinc micronutrient significantly improved plant height between 57.3 and 59.8%, while those with iron increased between 37.0 and 48.0% compared with water-irrigated plants. The leaf diameter was significantly influenced by the same treatments as plant height, treatments with zinc micronutrient (Zn Bac and Zn NPs + P Bac) increased the leaf diameter between 79.6 to 81.1%, and iron treatments (Fe NPs + P Bac, Fe Prec + P Bac, and Fe Bac) between 57.6 to 69.2%. Additionally, the biofertilizer treatment alone (P Bac) also exerted a significant effect on the diameter of the leaves, improving this parameter by 69.9%. With respect to root length, the experimental data did not lead to conclusive results. Statistically, this parameter presented high variability, which may be due to their size, which made it difficult to measure changes in the development of both primary and lateral roots due to the dimensions of the seedbed cavities, and tangled root structures that were handled carefully to prevent loss of plant material, which could impact weighing. Finally, the fresh weight of broccoli microgreens was significantly increased by the same treatments with zinc (150.4–178.0%), iron (112.1–156.1%), and the biofertilizer alone (123.9%), with respect to untreated plants (Figure 2).

### 3.2. Glucosinolates Profile in Broccoli Microgreens after the Application of Bio- and Nanofertilizers

**Figure 3:**
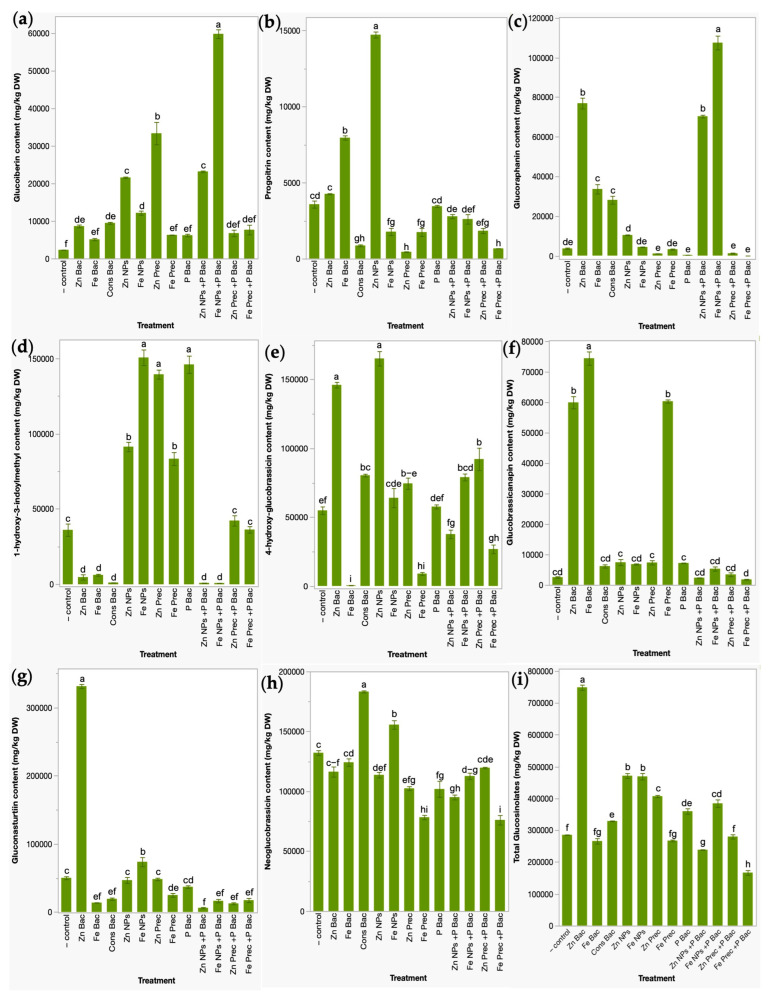
Effects of bio- and nanofertilizers on the contents of glucoiberin (**a**), progoitrin (**b**), glucoraphanin (**c**), 1-hydroxy-3-indoylmethyl (**d**), gluconasturtiin (**e**), 4-hydroxy-glucobrassicin (**f**), glucobrassicanapin (**g**), neoglucobrassicin (**h**), and total glucosinolates (**i**) in broccoli microgreens. Values correspond to means ± standard deviation. Values with different letters in the same column denote statistical differences between the mean of the treatments using Tukey’s test (*p* < 0.05). − control, water irrigated plants. Zn Bac, bacteria functionalized ZnO NPs. Fe Bac, bacteria capped γ-Fe_2_O_3_ NPs. Cons Bac, bacterial consortia used as capping agent. Zn NPs, uncapped ZnO NPs. Fe NPs, uncapped γ-Fe_2_O_3_ NPs. Zn Prec, mineral ZnO NP precursor. Fe Prec, mineral γ-Fe_2_O_3_, NP precursor. P Bac, phosphate-solubilizing bacterial biofertilizer. Zn NPs + P Bac, uncapped ZnO NPs applied with the biofertilizer. Fe NPs + P Bac, uncapped γ-Fe_2_O_3_ NPs applied in conjunction with biofertilizer. Zn Prec + P Bac, mineral ZnO NP precursor applied with biofertilizers. Fe Prec + P Bac, mineral γ-Fe_2_O_3_ NP precursor applied in combination with biofertilizer.

Eight glucosinolates were identified in the broccoli microgreen lyophilized powders (Figure 3), including four aliphatic glucosinolates, glucoiberin (GIB), progoitrin (PRO), glucoraphanin (GRA), and 1-hydroxy-3-indoylmethyl (1H3IM); one aromatic glucosinolate, gluconasturtiin (GNS); and three indolyl glucosinolates, 4-hydroxy-glucobrassicin (4HGBS), glucobrassicanapin (GBN), and neoglucobrassicin (NGBS). GIB was significantly improved by up to 25-fold with the uncapped γ-Fe_2_O_3_ NPs applied in conjunction with biofertilizer (Fe NPs + P Bac); other treatments also enhanced GIB concentrations including mineral ZnO NP precursor (Zn Prec;13-fold), uncapped ZnO NPs applied with the biofertilizer (Zn NPs + P Bac; 9-fold), uncapped ZnO NPs (Zn NPs; 8-fold), uncapped γ-Fe_2_O_3_ NPs (Fe NPs; 4-fold), bacterial consortia used as the NP capping agent (Cons Bac; 3-fold), and bacteria functionalized ZnO NPs (Zn Bac; 3-fold). The PRO content was only influenced by uncapped ZnO NPs and bacteria-capped γ-Fe_2_O_3_ NPs (3 to 1-fold); other treatments did not significant or negatively impact its concentration with respect to untreated plants. 

GRA was significantly increased with the uncapped γ-Fe_2_O_3_ NPs applied in combination with biofertilizer (Fe NPs + P Bac), bacteria-treated ZnO NPs (Zn Bac), uncapped ZnO NPs applied with the biofertilizer (Zn NPs + P Bac), bacteria capped γ-Fe_2_O_3_ NPs (Fe Bac), and bacterial consortia used as NP capping agent (Cons Bac), which increased its content by up to 29-fold, 20-fold, 18-fold, 8-fold, and 7-fold, respectively. 1H3IM concentrations were positively influenced (3 to 1-fold) by five treatments, including Fe-NPs, P Bac, Zn Prec, Zn NPs, and Fe Prec. Microgreens improved the 4HGBS content with uncapped ZnO NPs (201.01%), bacteria functionalized ZnO NPs (166.07%), mineral ZnO NP precursor applied with biofertilizer (67.90%), bacterial consortia used as the NP capping agent (46.53%), and uncapped γ-Fe_2_O_3_-NPs applied in conjunction with biofertilizer (43.98%). Regarding GBN, it was positively affected only with the bacteria capped γ-Fe_2_O_3_-NPs, mineral γ-Fe_2_O_3_-NP precursor, and bacteria functionalized ZnO NPs that increased GBN content by 28-fold, 23-fold, and 22-fold, respectively. The concentrations of GNS were improved with bacteria-treated ZnO NPs (560.60%) and uncapped γ-Fe_2_O_3_ NPs (46.96%). NGBS exclusively improved with two treatments, the bacterial NP capping agent and the uncapped γ-Fe_2_O_3_ NPs, which increased the NGBS content between 17.75 and 38.64%. Finally, the total glucosinolates were significantly increased by the following treatments: Zn Bac (162.14%), Zn NPs (65.07%), Fe NPs (64.29%), Zn Prec (42.52%), Fe NPs + P Bac (34.64%), P Bac (25.99%), and Cons Bac (15.22%).

### 3.3. Effects on the Concentration of Zinc and Iron Micronutrients in Broccoli Microgreens

**Figure 4:**
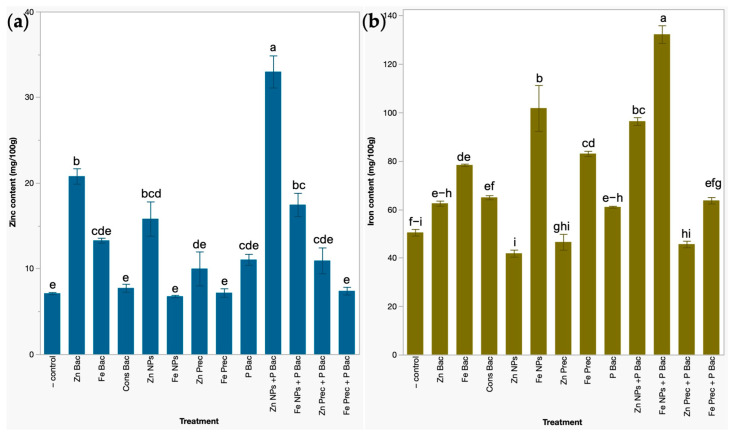
The contents of zinc (**a**) and iron (**b**) micronutrients in broccoli microgreens under bio- and nanofertilizer treatments. Results correspond to mean ± standard deviation; different letters above the error bars indicate significant differences between treatments by Tukey’s test at *p* < 0.05. − control, water irrigated plants. Zn Bac, bacteria functionalized ZnO NPs. Fe Bac, bacteria capped γ-Fe_2_O_3_ NPs. Cons Bac, bacterial consortia used as the capping agent. Zn NPs, uncapped ZnO NPs. Fe NPs, uncapped γ-Fe_2_O_3_ NPs. Zn Prec, mineral ZnO NP precursor. Fe Prec, mineral γ-Fe_2_O_3_ NP precursor. P Bac, phosphate-solubilizing bacterial biofertilizer. Zn NPs + P Bac, uncapped ZnO NPs applied with the biofertilizer. Fe NPs + P Bac, uncapped γ-Fe_2_O_3_ NPs applied in conjunction with biofertilizer. Zn Prec + P Bac; mineral ZnO NP precursor applied with biofertilizers. Fe Prec + P Bac, mineral γ-Fe_2_O_3_ NP precursor applied in combination with biofertilizer.

Bio and nanofertilizers were also applied to biofortify zinc and iron micronutrients in broccoli microgreens. In this sense, uncapped ZnO NPs applied together with the biofertilizer treatment (Zn NPs + P Bac), bacteria-capped ZnO NPs (Zn Bac), uncapped γ-Fe_2_O_3_ NPs applied with biofertilizer (Fe NPs + P Bac), and uncapped ZnO NPs (Zn NPs) positively affected the concentrations of zinc in broccoli microgreens by 363.41%, 191.99%, 145.95%, and 122.19%, respectively, compared with the untreated broccoli microgreens, where a concentration of 7.12 mg/100g of lyophilized powder was detected (Figure 4a).

The iron content was also significantly enhanced with the bio- and nanofertilization treatments; uncapped γ-Fe_2_O_3_ NPs applied in combination with biofertilizer (Fe NPs + P Bac) and uncapped γ-Fe_2_O_3_ NPs (Fe NPs) markedly increased iron concentrations by up to 161.57% and 101.71%, respectively, followed by uncapped ZnO NPs applied with the biofertilizer treatment (Zn NPs + P Bac), mineral γ-Fe_2_O_3_ NP precursor (Fe Prec). and bacteria functionalized γ-Fe_2_O_3_-NPs (Fe Bac), which improved the iron content by 91.00, 64.49, and 55.19%, respectively (Figure 4b).

## 4. Discussion

Dietary supplementation with beneficial, nutritious, and sustainably produced new food products has been recognized as an important measure to improve nutrition and health status and prevent and treat important diseases like obesity [17]. Therefore, bio- and nanofertilization practices were implemented in this work to biofortify broccoli microgreens with relevant phytochemicals and micronutrients. Regarding their impact on plant growth, the treatments with the best effects in the four agronomic parameters evaluated were bacteria-functionalized ZnO NPs (Zn Bac), uncapped ZnO NPs applied in conjunction with the biofertilizer treatment (Zn NPs + P Bac), uncapped γ-Fe_2_O_3_ NPs applied with biofertilizer (Fe NPs + P Bac), mineral γ-Fe_2_O_3_ NP precursor applied in combination with biofertilizer (Fe Prec + P Bac), bacteria capped γ-Fe_2_O_3_ NPs (Fe Bac), and the biofertilizer treatment alone (P Bac). Other studies have also reported the positive influence of zinc [49,50,51] and iron [51,52] nanoparticles, as well as the bacterial species present in the P Bac consortia [27] on the growth parameters of broccoli. Awan et al. [49] found that the application of ZnO NPs (24 nm) increased the seed germination (37.5%), root length (56.6%), shoot length (16.6%), and weight (41%) of broccoli seedlings. As in this work, they also identified that zinc oxide nanoparticles demonstrated a higher efficiency for improving crop growth than the macro size salt precursor solution. Similarly, Farhan et al. [52] observed that using nano iron at 50 ppm significantly improved plant height, leaf number, leaf area, plant yield, root weight, and iron concentration in leaves. Concerning the biofertilizer treatment (P Bac), it was previously evaluated in another study where the beneficial PGPM present in this consortium was isolated and characterized. In that work, P Bac significantly increased the plant height, leaf diameter, and fresh weight of broccoli microgreens by 41.51, 63.66, and 66.20%, respectively [27].

Interestingly, this study suggests synergistic interactions between bio- and nanofertilization practices, that is, the application of nanoparticles in conjunction with plant growth-promoting microorganisms, as neither the nanoparticles nor the mineral precursors alone exerted significant effects with respect to the control. The treatments consisting of nanoparticles subjected to the functionalization process with a consortium of *Pseudomonas* species (Zn Bac, and Fe Bac) and those involving uncapped NPs applied together with the biofertilizer treatment (Zn NPs + P Bac, and Fe NPs + P Bac) both deliver nanoscale micronutrients and viable beneficial microorganisms to the plants. It has been reported that, in the combined application, nanoparticles can promote the growth of PGPM, improve the enzymatic microbial activity, and enhance their beneficial effects on plants [39,53,54,55]. At the same time, PGPM optimizes nutrient absorption, increasing the nanoparticles’ stability and bioavailability [49]. Seyed Sharifi et al. [54] evaluated the impact of zinc and iron oxide nanofertilizers and biofertilizers *(Azotobacter, Azosperilium*, and *Pseudomonas*) on the physicochemical properties and grain yield of wheat (*Triticum aestivum* L.) under water limitation conditions. They found that the application of biofertilizers and nanoparticles increased the proline content, photosynthetic pigments, soluble sugars, and enzyme activities; particularly, the treatment with *Azotobacter* and nano Zn−Fe oxide enhanced grain yield by 88% compared with the control under severe water limitations, concluding that the combined application was more successful in improving plant yield as compared to the individual application of each fertilizer. In another study, Singhal et al. [55] demonstrated that ZnO nanorods applied at 500 ppm significantly increased the biomass of the beneficial fungus *Piriformospora indica* DSM 11827P, which resulted in a synergistic association that enhanced the biomass productivity of broccoli.

Concerning glucosinolate production, depending on the glucosinolate, this was enhanced by applying micronutrients, PGPM, or their combination. Zinc and iron micronutrients perform vital functions for normal plant development and metabolism, having a significant influence on hormone biosynthesis and regulation, chlorophyll and carbohydrates production, protein synthesis, photosynthesis, DNA stability/repair, membrane function, and enzyme activation [1,54]. They are essential micronutrients that are key for over 300 enzymes and hormones. Thus, it is inferred that when the micronutrient availability and uptake are increased, the accumulation of certain phytochemicals and secondary metabolites, including glucosinolates, is improved due to enhanced plant metabolism and nutrient status [49]. GLSs are classified according to their precursor amino acids: aliphatic GLSs are built from alanine, leucine, isoleucine, methionine, or valine; aromatic GLSs are derived from phenylalanine or tyrosine; and indole GLSs originate from tryptophan [12,56]. Micronutrients can improve the content of amino acid precursors of glucosinolates, e.g., zinc content can improve the concentrations of L-tryptophan, which can increase the biosynthesis of indole glucosinolates [57]. It is also reported that Zn toxicity can cause greater accumulation of GLSs, triggered by pathogen-resistance-related genes as a response to a metal-stress-derived signal. However no toxic symptoms were identified in the broccoli microgreens [58].

Beneficial microorganisms can also impact glucosinolate production by triggering the plant’s defense mechanisms though systemic resistance. Glucosinolates are involved in the plant response against plant tissue damage or microbial pathogen attack [21,56,59,60]. The synthesis of aliphatic, aromatic, and indole glucosinolates varies depending on the environmental stressors or microbe species [60]. Little is known about the specific interaction between individual glucosinolate contents and the colonization of plant growth-promoting bacteria, but it is suggested that the induction of the plant’s systemic resistance can result in physiological, biochemical, and metabolic changes that lead to the synthesis of secondary metabolites, including glucosinolates, required in plant defense mechanisms. The major compounds responsible for the PGPR-mediated induced systemic resistance (ISR) include lipopolysaccharides, lipopeptides, pyocyanin, siderophores, antibiotics, iron-regulated compounds, bacterial quorum sensing molecules, volatile 2,3-butanediol, and N-alkylated benzylamine [1,59,61,62]. PGPR performs this process to prepare plants before the pathogen’s attack and reduce the incidence of disease or damage. ISR is then a state of enhanced defensive capacity carried out by a plant that has responded to a specific biotic or chemical stimulant [59,62]. PGPM can also influence plant hormone levels, including jasmonic acid (JA) and salicylic acid (SA), which are involved in plant defense responses and have been implicated in glucosinolates production [58].

Enhancing glucosinolate contents in plants has attracted important pharmacological interest due to their effects on promoting human health, mainly their anticarcinogenic, antiobesity, anti-inflammatory, and antimicrobial properties. Applications of bio- and nanofertilizers as elicitors may be an effective option to increase desired glucosinolates [20,60]. Glucosinolate function is mostly associated with their hydrolysis products, which are generated by thioglucoside glucohydrolase, also known as myrosinase, which hydrolyzes the glucose moiety of the core glucosinolate structure, releasing glucose and an unstable aglycone compound that can be transformed to form isothiocyanates, nitriles, thiocyanates, epithionitriles, and oxazolidines, depending on the starting glucosinolate structure [21,56]. Hydrolysis products are relevant for the plant defense against insects, bacteria, and fungi [56].

Micronutrient deficiencies in people are linked to micronutrient deficits in plants [24]. Thus, considering the increasing rates of micronutrient deficiencies, mainly zinc and iron, research directed to improve food quality is of major relevance. The biofortification of micronutrients by applying nanoparticles has been widely studied in different food crops, including rice, wheat, maize, broccoli, and chickpea [52,63,64,65,66]. In the present study, treatments consisting of uncapped ZnO and γ-Fe_2_O_3_ NPs, nanoparticles functionalized with PGP bacterial consortium (Zn Bac, and Fe Bac), and uncapped NPs applied with a biofertilizer treatment (Zn NPs + P Bac, Fe NPs + P Bac) were the most important to improve zinc (145.95–363.41%) and iron (55.19–161.57%) contents in broccoli microgreens. Sundariae et al. [66] applied iron oxide nanoparticles to biofortify wheat (*Triticum aestivum* L.) crops through seed priming, increasing the grain iron contents between 26.8 to 45.7%. Yang et al. [64] improved the Zn concentration of brown rice between 13.5 to 39.4% by applying ZnO NPs compared with conventional fertilization. Du et al. [65] also evaluated zinc nanofertilization on wheat (*Triticum aestivum* L.), they identified that ZnO NPs were more effective than salt NP precursors (ZnSO_4_) at increasing the grain Zn content, but, particularly, they found that ZnO NPs were not detected in the wheat tissues, attributing this phenomenon to the dissolution of ZnO NPs in the rhizosphere and the plant absorption and transport of Zn in the ionic form. Similar effects have been obtained by different authors [67,68,69], and may be one of the starting mechanisms for zinc and iron biofortification.

Metal-based nanoparticles like ZnO and γ-Fe_2_O_3_ NPs can be subjected to different surface interactions and biochemical transformations in the soil-root interface, which depend on the characteristics of the nanomaterials such as capping agents, size, and charge [70]. Biochemical transformations may involve the release of zinc or iron ions (Zn^2+^, Fe^2+^ or Fe^3+^) from the nanoparticles into the soil; this process is mediated by factors like soil pH, redox conditions, and the contact with root exudates (organic acids, such as gluconic and citric acid, released by plant roots and microorganisms participate in the dissolution of nanoparticles). For example, the rhizosphere can be acidified by the H-ATPase AHA2, which extrudes protons from the root and can solubilize Zn and Fe through cation exchange in the rhizosphere, Fe^3+^ is reduced to ferrous Fe^2+^ by ferric chelate reductase (FRO2) and is absorbed by root epidermal cells by the metal carrier IRT1 (plasma membrane-localized Fe^2+^ transporter), while Zn is absorbed mainly through transmembrane transporters in the ZIP (ZRT and IRT-like protein) family that is localized in the membrane of root epidermal cells [25]. The rhizosphere is a biologically and chemically active zone enriched with microorganisms and root exudates that can promote the biotransformation of nanoparticles prior to their absorption [67,70,71].

The transformation of NPs can also occur inside the plant tissues, resulting in various accumulated elemental speciation in plants [70,72]. In general terms, when the nanoparticles are not dissolved in the rhizosphere, the mechanism of absorption and translocation of soil-applied nanoparticles starts in the root surfaces and root-damaged sites where these compounds interact with the epidermal cells to penetrate through apoplastic and symplastic pathways and reach the vascular system, they are then translocated to the shoot via xylem vessels, depending on the charge of the nanoparticle they are more easy (negatively charged particles) or difficult (positive or neutral charge particles) to be transported. NPs are finally accumulated in the aerial plant organs (vacuoles and cell walls are important sites for accumulation), where they can be transformed into ionic forms [1,73,74].

In addition to nanoparticles, another important factor contributing to the zinc and iron biofortification in the broccoli microgreens is the presence of PGPM in the most relevant treatments. Bacterial isolates used in this study were previously characterized for several PGP traits, showing a potential mineral solubilizing activity (including phosphate, potassium, and zinc) and siderophores production [27]. The main mechanism of zinc solubilization is through the reduction in pH in the rhizosphere; for this purpose, microorganisms produce organic acids, including citric, gluconic, acetic, lactic, formic, malic, and oxalic acids, to acidify the rhizospheric soil. Small changes in the soil pH significantly promote the release of bioavailable Zn forms in the soil [1]. Acidic environments also promote the dissolution of nanoparticles [70]. Species of the genera *Serratia* and *Pseudomonas* have been reported in different studies to have important effects for promoting plant growth and biofortifying crops with zinc [75,76]. Regarding siderophore production by beneficial microorganisms, these compounds also increase zinc and iron bioavailability. Siderophores like bacillibactins, pyoverdines, and cephalosporins chelate insoluble zinc and iron forms; they can scavenge Zn^2+^ and Fe^3+^ from the mineral phases and generate soluble Zn^2+^- and Fe^3+^-siderophores complexes that are absorbed into plant cells, also promoting the solubilization of the nanoparticles and facilitating the uptake of ionic forms [1,70].

Finally, regarding the recommended dietary intake of broccoli microgreens, and considering the reference daily intake (RDI) for zinc (8–11 mg/day) and (8–18 mg/day) in adults of 19 years old and older, an individual would have to consume 230–315 g/day and 59–132 g/day of biofortified fresh broccoli microgreens to reach the recommended daily dose of zinc and iron, respectively. Estimates were made considering an average moisture content of 90% for broccoli microgreens, and the highest concentrations of zinc (34.87 mg/100 g DW) and iron (135.9 mg/100 g DW) obtained in this work [77].

## 5. Conclusions

This work demonstrated the potential of utilizing ZnO and γ-Fe_2_O_3_ nanofertilizers combined with biofertilizers based on native plant growth-promoting bacteria to enhance plant growth and biofortify micronutrients and glucosinolates in broccoli microgreens, identifying possible synergistic interactions between nanoparticles and PGPR to improve the growth and nutritional quality of microgreens. Based on the agronomic growth parameters, the most relevant treatments were nanoparticles functionalized with PGP-bacterial consortium (Zn Bac, and Fe Bac), and uncapped NPs applied with a biofertilizer treatment (Zn NPs + P Bac, and Fe NPs + P Bac); moreover, these treatments and the uncapped ZnO and γ-Fe_2_O_3_ NPs also exerted significant increases in the glucosinolates and zinc and iron contents. Further experiments will be performed to evaluate their effect in other important food crops, as well as in the later stages of plant development. This study validated that applying zinc and iron nutrients as nanofertilizers combined with PGPR as biofertilizers is an important strategy for the rapid and efficient delivery of nutrients into the plant system, the enhancement of plant nutrient uptake to fulfill the plants’ requirements, and the improvement of plant’s nutritional value and growth within a short period of time.

## Data Availability

The data used to support the findings of this study can be made available by the corresponding author upon request.
